# Gene expression profiling identifies activated growth factor signaling in poor prognosis (Luminal-B) estrogen receptor positive breast cancer

**DOI:** 10.1186/1755-8794-2-37

**Published:** 2009-06-24

**Authors:** Sherene Loi, Christos Sotiriou, Benjamin Haibe-Kains, Francoise Lallemand, Nelly M Conus, Martine J Piccart, Terence P Speed, Grant A McArthur

**Affiliations:** 1 Department of Research, Molecular Oncology Lab, Peter MacCallum Cancer Centre, East Melbourne, Victoria, Australia; 2 Translational and Functional Genomics Unit, Jules Bordet Institute, Brussels, Belgium; 3 Department of Medicine, St. Vincent Hospital, University of Melbourne, Victoria, Australia; 4 Department of Bioinformatics, Walter and Eliza Hall Institute, Parkville, Victoria, Australia

## Abstract

**Background:**

Within estrogen receptor-positive breast cancer (ER+ BC), the expression levels of proliferation-related genes can define two clinically distinct molecular subtypes. When treated with adjuvant tamoxifen, those ER+ BCs that are lowly proliferative have a good prognosis (luminal-A subtype), however the clinical outcome of those that are highly proliferative is poor (luminal-B subtype).

**Methods:**

To investigate the biological basis for these observations, gene set enrichment analysis (GSEA) was performed using microarray data from 246 ER+ BC samples from women treated with adjuvant tamoxifen monotherapy. To create an *in vitro *model of growth factor (GF) signaling activation, MCF-7 cells were treated with heregulin (HRG), an HER3 ligand.

**Results:**

We found that a gene set linked to GF signaling was significantly enriched in the luminal-B tumors, despite only 10% of samples over-expressing HER2 by immunohistochemistry. To determine the biological significance of this observation, MCF-7 cells were treated with HRG. These cells displayed phosphorylation of HER2/3 and downstream ERK and S6. Treatment with HRG overcame tamoxifen-induced cell cycle arrest with higher S-phase fraction and increased anchorage independent colony formation. Gene expression profiles of MCF-7 cells treated with HRG confirmed enrichment of the GF signaling gene set and a similar proliferative signature observed in human ER+ BCs resistant to tamoxifen.

**Conclusion:**

These data demonstrate that activation of GF signaling pathways, independent of HER2 over-expression, could be contributing to the poor prognosis of the luminal-B ER+ BC subtype.

## Background

Gene expression profiling has given new insight into the heterogeneity of estrogen receptor positive breast cancers (ER+ BC). In particular, much interest has focused on predicting resistance in tamoxifen-treated patients, given the emergence of alternative anti-estrogen therapies and targeted biological agents [1]. There are several predictive gene sets and assays that have been developed to identify groups of ER+ BC that have a poor clinical outcome despite tamoxifen treatment [2-6]. Previously, by using a meta-analytical approach in over 2800 breast cancers, we have shown that proliferation and cell cycle-related genes are the most powerful predictors of prognosis in breast cancer and seems to be the common biological signal linking many of these prognostic predictors, despite the different methods of discovery and lack of significant gene overlap [6-8]. Using a quantitative proliferation index called a Grade Gene-expression Index (GGI- genomic grade) [8], we were able to divide ER+ BC according to their cellular proliferative activity into two prognostic groups associated with statistically distinct clinical outcome [6]. These subgroups are highly correlated to other commonly cited molecular classifications of breast cancer: the luminal A/B classification [9] and the good and poor prognostic subgroups produced by the 21-gene Recurrence Score [4,6] similar to the low and high genomic grade ER+ BC subtypes respectively.

However, whilst many gene classifiers exist for predicting prognosis in women with ER+ BC treated with tamoxifen, there has been little focused biological investigation into the mechanisms that could be contributing to the relative tamoxifen resistance and the increased proliferative activity in the high risk, luminal-B ER+ BC subtype. Elucidation of the relevant dysfunctional pathways would ultimately lead to new strategies to treat the poor prognosis subgroup and may eventually prove a more clinically relevant way to stratify treatment options for breast cancer patients. Whilst gene expression data has demonstrated its ability to identify subsets of disease, predict disease outcome or response to therapeutics [9-13], extracting useful biological data from whole genome microarray data to identify new therapeutic approaches in the clinical setting remains a significant challenge. Recently, a new computational method of analysis known as gene-set enrichment analysis (GSEA) [14] has provided a new approach of identifying potentially relevant oncogenic pathways that may be correlated with particular phenotypes. This pathway orientated approach has advantages over the individual gene analyses which can often fail to put genes into context and can be difficult to reproduce across different datasets. This concept was originally demonstrated in a study which identified a set of genes involved in oxidative phosphorylation, whose expression was concurrently decreased in human diabetic muscle when no single gene was significant by classical analysis, emphasizing the importance of elucidating pathway relationships [15]. However GSEA still remains hypothesis generating and requires experimental validation. Bearing this in mind, we report the use of GSEA to generate hypotheses about dysfunctional biological pathways that could be driving proliferation and contributing to the poor outcome of the tamoxifen-treated, highly proliferative (luminal-B) ER+ BC subtype.

## Methods

### Human breast cancer microarray dataset

Our current analysis uses a microarray dataset of 246 early- stage breast cancer samples, all of whom were classified as estrogen receptor (ER) positive and had received adjuvant tamoxifen monotherapy only. These samples have been previously used in another study (methods and demographics are described in Loi *et al*. [6] with the raw data available at the Gene Expression Omnibus (GEO) repository database , accession code GSE6532. Microarray analysis was performed with Affymetrix™ U133A Genechips^® ^(Affymetrix, Santa Clara, CA) according to Affymetrix™ protocols. There were 99 tumors classified as highly proliferative (high-risk, luminal-B ER+ BC subtype) tumors and 147 lowly proliferative (low-risk, luminal-A ER+ BC subtype) tumors used for this analysis. Each hospital's institutional ethics board approved the use of the tissue material for research purposes.

Data was also analyzed through the use of Ingenuity Pathways Analysis (; Ingenuity Systems, Redwood City, CA) version 6.0.

### Gene set enrichment analysis (GSEA)

Gene set enrichment analysis was performed using the GSEA software version 1.0 developed by Subramanian and colleagues at the Broad Institute [14]. For this analysis we compared low and high-risk ER+ subtypes and looked for enrichment of the C2 curated catalogue of functional genes sets. We also curated from the literature gene sets representing ERBB2/growth factor activation from Perou et al (see Additional file 1) [9], androgen receptor [16], *RAS, MYC SRC, E2F3 *and β-Catenin [17] oncogenic pathway dysregulation. 1000 permutations were used to test significance of enrichment.

In brief, GSEA is a supervised method of elucidating differential expression (enrichment) of gene sets from gene expression data. These sets contain genes that share or relate to a common biological function and are determined *a priori*. The computational method ranks all genes in the data set based on their correlation with the two phenotypes being compared (in this case low vs. high-risk ER+ BC subtypes). It then identifies the rank positions of the members of the specified gene set in relation to either phenotype and calculates an enrichment score (ES) which reflects the degree to which a gene set is over represented at the extremes. The class labels are then permutated to compare the ES obtained by the specific gene set to that obtained across random classes to calculate a p value for enrichment. To control for multiple hypothesis testing, a false discovery rate (FDR) is calculated to control the proportion of false positives.

### Cell Culture and treatment

MCF-7 and SK-BR3 cells were grown at 37°C, 5% CO_2 _in phenol-red free Improved Modified Eagle's medium (IMEM) supplemented with 5% fetal bovine serum. Cells were plated in 100 mm dishes and grown to 80% confluence. To create an *in vitro *model the highly proliferative ER+ subtype with growth factor (GF) pathway activation, MCF-7 cells were treated with heregulin-beta 1 [HRG- CytoLab/Peprotech Asia] [18]. Before treatment, cells were serum starved for 24 hours. Cells were then treated with vehicle (ethanol), estrogen [E2- Sigma] (1 nM), tamoxifen [TAM] (4-hydroxy-tamoxifen-Sigma, 100 nM), heregulin-beta 1 [HRG- CytoLab/Peprotech Asia] (10 ng/mL) or tamoxifen and heregulin [TAM-HRG] (same concentrations) for 24 hours (the cells treated for western blot analysis were incubated for 20 minutes in the presence of the relevant stimulus) prior to collection.

### Cell cycle analysis

Cells described above were collected for cell cycle analysis. Cells were analyzed using flow cytometric analysis. Cells were labeled with Bromodeoxyuridine (BrdU, Sigma) and then fixed and stained with bromodeoxyuridine-conjugated FITC and propidium iodide. BrdU was detected with anti-BrdU (Becton Dickinson) and cell cycle analysis was carried out using a Becton Dickinson FACScan.

### Antibodies

Antibodies against ERB2 (# 2242), ERB3 (# 4754), phosphorylated ERB2 (Tyr877; # 2241), ERB3 (Tyr1289; # 4791), tyrosine (P-Tyr-100; # 9411), AKT (S473; # 9271, S6 (S235/236; # 2211) and ERK (T202/Y204; # 9101) were obtained from Cell Signaling and β actin from MP Biomedicals. Cell Signaling antibodies were used at 1:1000 dilution and β actin at 1:20,000.

### Immunoblots

After treatment, cells were rinsed in PBS and lysed for 20 min in ice cold 1% NP40 lysis buffer (50 mM Tris-HCl pH 7.5, 120 mM NaCl, 1 mM EDTA, 50 mM NaF, 40 mM β-glycerophosphate, 0.1 mM Na_3_VO_4_, 1 mM benzamidine, 1% NP40,1 mM PMSF, 1 protease inhibitor cocktail tablet – Roche –/10 ml lysis buffer). Cellular debris was removed by 2 × 10 min centrifugation at 4°C. Protein concentration was determined in the supernatants by Lowry (BioRad kit) and aliquots of 50 (g proteins were separated in reducing denaturing conditions by standard SDS-PAGE gel electrophoresis on 10% gels. SK-BR-3 cell lysates (+/- HRG) were run on each gel as controls for HER2 overexpression. Blotting of proteins, incubation with antibodies and protein detection was done as previously described using the LiCor Odyssey infrared Imaging System (Biosciences) [19]. Cell lysates were collected

### Clonogenic survival assays

MCF-7 cells were plated at 2000 cells per 60 mm plate. Twenty-four hours later, cells were treated with drugs at the specified doses and incubated in normal medium containing drug for 12 days, after which they were fixed in 90% methanol, and stained with 0.1% crystal violet. Colonies of >50 cells were enumerated.

### Anchorage independent growth assays

The ability of HRG treated MCF-7 cells to stimulate anchorage-independent growth in the presence of TAM was tested by soft agar assay. Colony forming assays in soft agarose were performed. In brief, 4000 cells (suspended in IMEM phenol-red free medium supplemented with 5% fetal bovine serum) in 0.35% bactoagar with different treatments were grown on the bottom layer of solidified 0.6% bactoagar in 35 mm dishes. After 10 days the colonies on the top layer were enumerated. The size exclusion limit for a positive colony counting was ~60 μm diameter.

### Microarray experiments of cell lines

To determine if the ERBB2/growth factor activation gene set was enriched in an *in vitro *model of the high-risk ER+ subtype, MCF-7 cells that were serum starved for 24 hours by culturing the cells in IMEM containing 0.1% serum. Cells were then stimulated with HRG before RNA was extracted using TriZOL (Invitrogen) and compared with untreated serum starved MCF-7 cells. Microarray analysis was performed according to standard Affymetrix protocols: biotinylated targets were prepared by published methods (Affymetrix, Santa Clara, CA, USA) and hybridized to the Affymetrix oligonucleotide microarray U133 Plus GeneChip^®^. Arrays were scanned using standard Affymetrix protocols. Gene expression values from the CEL files were normalized by use of the standard quantile normalization method in RMA [20]. Gene expression profiles were then uploaded into GSEA and analyzed for enrichment (MCF untreated controls vs. MCF-7 cells treated with HRG).

Proliferation status of these cell lines was assessed and compared by calculating their "Grade Gene expression Index" (GGI) as previously described [8] and by assessing for significance of cell cycle related gene sets. In this way we could determine if the MCF-7 cells treated with HRG had a higher GGI, i.e. higher expression of proliferation genes compared with untreated MCF-7 cells. Cell samples corresponding to each treatment were arrayed in triplicate.

### Statistical Analysis

Values are expressed as means with 95% confidence intervals (CIs). The effects of drug treatment (TAM, HRG, HRG+TAM) on MCF-7 cells were compared by the % S phase of serum-starved cells using a two sample t test. P ≤ 0.05 was set as the criteria for statistical significance. Experiments were done in triplicate and repeated three times.

## Results

### GSEA analysis of high-risk (luminal-B) and low (luminal-A) ER+ BC subtypes

Previously we have reported that a ER+ BC subtype defined by high levels of expression of proliferation genes has a poor outcome on adjuvant tamoxifen monotherapy by analyzing 249 archival tissue samples from women diagnosed with early-stage breast cancer [6]. We applied GSEA to identify gene sets correlated with the poor prognosis ER+ BC subtype. Using a p value of <0.05 and false discovery rate (FDR) of ≤ 0.20, there were 78 significant gene sets (Additional file 2). Many of these gene sets were, as expected, related to cell cycle function. Interestingly, there were a number of gene sets related to metabolic and apoptosis pathways. We were also interested to note the significance of the ERBB2 gene set (p = 0.02, FDR = 0.09), given that only a minority of these samples over-expressed HER2 by immunohistochemisty (+3). After excluding these samples (10%) we found that this gene set remained significantly enriched in the high risk ER+ BC subtype (p < 0.001, FDR = 0.01). It is known that overexpression of HER2 leads to tamoxifen resistance [21] but as the ERBB2 gene set could also represent signaling in response to any other growth factor, we hypothesized that it was activation of the downstream mediators of this pathway that could be playing an important role in contributing to the poor prognosis molecular subtype's phenotype of high proliferation and tamoxifen resistance. To further examine the biologic function of the ERBB2 gene set, we performed Ingenuity Pathway analysis (IPA). Neuregulin signaling was ranked the second top significant canonical pathway (p = 0.005) supporting the hypothesis that this gene set represented growth factor (GF) signaling (Additional file 3).

### HRG- treated MCF-7 breast cancer cells as a biological model for growth factor signaling pathway activation

**Figure 1:**
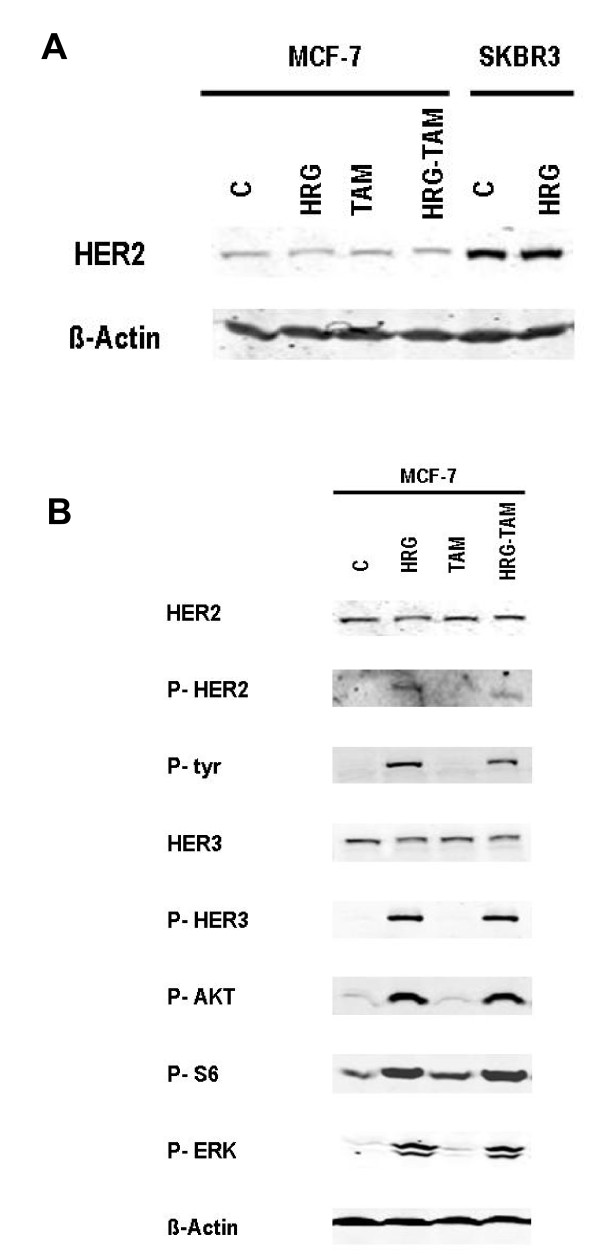
**Western blot analysis for HER2 and HER3 and phosphorylated- HER2 (P-HER2), HER3 (P-HER3), AKT (P-AKT), S6 (P-S6) and ERK (P-ERK) in serum starved MCF-7 cells treated with vehicle (C), tamoxifen [100 nM] (TAM), heregulin [10 ng/mL] (HRG) or tamoxifen and heregulin (HRG-TAM)**. **(A) **SK-BR3 cells were a positive control for HER2 overexpression. Compared with the SK-BR3 cells, the MCF7 cells do not overexpress HER2. **(B) **HRG alone or in combination with tamoxifen stimulates HER2, HER3, AKT, S6 and ERK phosphorylation as compared to vehicle and tamoxifen alone. These figures shows a representative blot from three separate western blots.

To create a model of growth factor (GF) pathway signaling activation, MCF-7 cells were treated with heregulin (HRG) [18]. MCF-7 cells normally express low or normal levels of HER2 and HER3 is found in HER2 immunoprecipitates when MCF-7 cells are treated with the HER3 ligand, HRG [18]. As seen in Figure 1a, MCF-7 cells treated with HRG do not over-express HER2 protein compared with SK-BR3 breast cancer cells that are known to contain amplified ERRB2. However, HRG stimulates phosphorylation of HER2 and HER3 at levels above untreated MCF-7 cells despite the lack of HER2 overexpression (Figure 1b). Phosphorylation of AKT, ERK/MAPK, and S6 were also induced by HRG, further supporting activation of GF signaling in response to stimulation by HRG.

### HRG-induced growth factor signaling results in resistance to tamoxifen in MCF-7 cells

**Figure 2:**
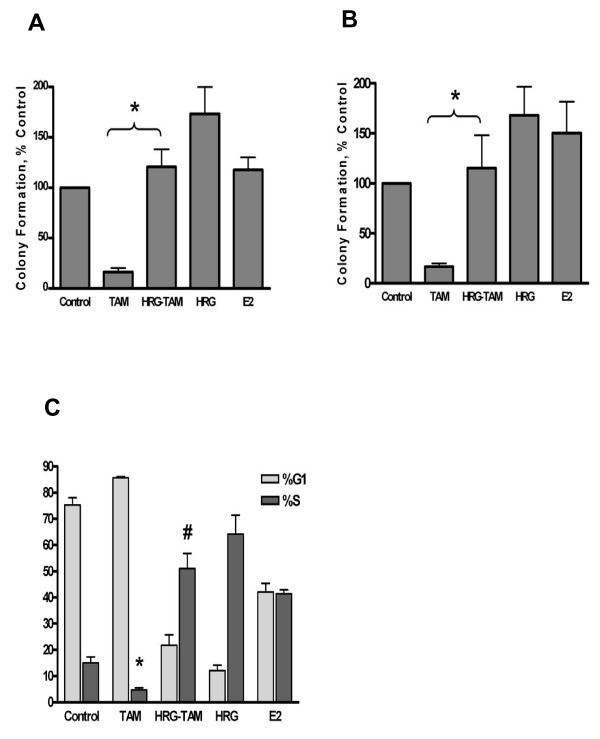
**Heregulin reverses the anti-proliferative effect of tamoxifen in clonogenic and anchorage independent assays**. **(A) Clonogenic assays**: MCF-7 cells cultured at low density in standard 60 mm tissue culture plates. Cells were treated with vehicle (control), estrogen [1 nM] (E2), tamoxifen [100 nM] (TAM), heregulin [10 ng/mL] (HRG) or tamoxifen and heregulin (HRG-TAM) and colonies enumerated after 12 days. *p < 0.001 comparing tamoxifen-treated and heregulin with tamoxifen treated cultures. **(B) Anchorage-independent growth assays**: MCF-7 cells in 0.35% bactoagar with the different treatments as above were grown on the bottom layer of 0.6% bactoagar in 35 mm tissue culture plates. Colonies were enumerated after 10 days. *P < 0.001 comparing tamoxifen-treated and heregulin with tamoxifen treatedcultures Graphs represent the mean colony number of nine dishes. Error bars indicate 95% CI. **(C)**: Serum starved MCF-7 cells were treated with vehicle (control), tamoxifen [100 nM] (TAM), heregulin [10 ng/mL] (HRG) or tamoxifen and heregulin (HRG-TAM) for 24 hours and analyzed by flow cytometry. Each column represents the mean of the percentage of cells in G1 and S phase of the cell cycle from triplicate plates; error bars indicate 95%CI; * p < 0.001 comparing TAM and control; # p < 0.001 comparing TAM and HRG-TAM cells.

Next, we sought to determine if our model of HRG-induced GF pathway activation could induce resistance to tamoxifen. Tamoxifen's effects on the growth of MCF-7 cells were assessed by clonogenic and anchorage independent growth assays. As expected, treatment of MCF-7 cells with estrogen increased both clonogenic (p = 0.01) and anchorage-independent colony formation (p = 0.001) and treatment with tamoxifen reduced it (p = 0002, p = 6.9 × 10^-12 ^respectively) (Figure 2a, b). However, treatment with HRG overcame tamoxifen's growth inhibitory effects on MCF-7 cells in both clonogenic (p = 6.7 × 10^-7^) and anchorage-independent (p = 1.4 × 10^-6^) assays. Together, these data demonstrate that our *in vitro *model of GF pathway activation, as evidenced by phosphorylation of HER2, HER3, ERK and S6 can overcome tamoxifen-induced cell cycle arrest in MCF-7 cells, whose growth are normally highly sensitive to tamoxifen treatment.

As estrogen can regulate the transition of breast epithelial cells from G0 into S-phase of the cell cycle, we also examined the effects of HRG on cell cycle arrest induced by TAM under conditions of low growth factors. Treatment of MCF-7 cells with TAM for 24 hours reduced the fraction of cells in S phase cells from 15.0% in cells that had been cultured without serum for 24 hours to 4.7% (p = 3.4 × 10^-6^). Treatment with HRG and E2 caused an increase in the % of cells in S phase under these conditions (p < 0.001 for both). Treatment of MCF-7 cells with both TAM and HRG caused the percentage of S phase cells to increase to 51.0% (p = 3.6 × 10^-8 ^when compared with TAM alone). This result was similar to increase in S-phase fraction observed following treatment with HRG alone (Figure 2c). Together, the results of these experiments support the hypothesis derived from the GSEA-analyzed human microarray data proposing that the activation of the downstream mediators of GF signaling contributes to tamoxifen resistance in ER+ BC.

### Gene expression profiling of HRG-treated MCF-7 cells identifies a similar expression profile to the highly proliferative (luminal-B) ER+ human breast cancer subtype

Previous microarray cluster analysis has shown that MCF-7 cells are characterized by high expression of genes associated with luminal epithelial cells, including the estrogen receptor and that this cell line is an acceptable model for the ER+/ERBB2- luminal tumors [22]. To examine whether treatment of MCF-7 cell lines with HRG results in enrichment of the ERBB2/GF signaling gene set and a highly proliferative phenotype compared with untreated MCF-7 cells, RNA was extracted for microarray analysis. Gene expression profiles were then uploaded into GSEA to determine if the ERBB2/GF signaling gene set was highly correlated with the HRG-treated MCF-7 cells. This was confirmed (p < 0.001, FDR = 0.16), supporting our immunohistochemistry protein phosphorylation results. Other gene sets supporting up-regulation of the cell cycle function were also significantly enriched in the HRG-treated MCF-7 cells (Additional file 4)

Finally, the quantitative assessment of the proliferation activity of HRG-treated MCF-7 cells was compared with untreated cells using the GGI. This analysis confirmed that the HRG-treated cells had higher GGI scores (p = 0.02). These data suggest that HRG-treated MCF-7 cells may be useful as an *in vitro *model of the luminal-B ER+ BC subtype.

## Discussion

In order to identify important underlying oncogenic pathways that could be contributing to the poor prognosis of the luminal-B, ER+ BC subtype, we have used gene set enrichment analysis (GSEA) to interrogate microarray data derived from human breast cancer to generate potential biological hypotheses. To our knowledge, this is the first study specifically investigating the biology of the luminal-B ER+ BC subtype. Here, we have shown that MCF-7 cells, which are normally highly sensitive to tamoxifen treatment *in vitro*, can, overcome tamoxifen-induced cell cycle arrest when treated with the HER3 ligand heregulin (HRG), which creates a scenario of growth factor pathway activation, supporting the GSEA-derived hypothesis. MCF-7 cells treated with HRG also have higher expression of proliferation genes correlating with significantly higher GGI levels. Furthermore, we subsequently confirmed enrichment of the growth factor signaling gene set in the HRG-treated MCF-7 cell lines which was associated with phosphorlyation of important downstream proteins commonly used to identify activation of this pathway.

Whilst we curated the growth factor signaling gene set from those genes associated with the ERBB2 over-expressing breast cancer subtype of Perou et al [9], it is possible that this set of genes can be expressed in response to activation of any other growth factor or activating mutation in the major signaling pathways downstream of growth factor receptors. Two recent studies however, have demonstrated the importance of ligand-induced signal amplification as an important mechanism of HER2 receptor and pathway activation in tumors with low HER2 levels [18,23]. Notably, MCF-7 cells are known to have low levels of HER2 protein expression. Tamoxifen resistance in ER+ BC is a major clinical problem and whilst mechanisms of inherent and acquired tamoxifen resistance are not fully understood, there is growing evidence of the significance of activation of receptor tyrosine kinase pathways. As HER2 is such an important kinase in breast cancer, it is not inconceivable that certain breast cancers with low overall HER2 expression but with many activated receptors are still highly dependent on this pathway for proliferation and growth. Our study provides evidence that activation of growth factor signaling could be a significant contributor to the luminal-B phenotype of ER+ BC. Further experiments to define if our observations are mediated through HER2 or other members of the EGFR family will be important to determine if targeting activated ERBB2 signaling in patients with the luminal-B phenotype could be of therapeutic benefit. A recent study reported that the gene expression profile associated with insulin-like growth factor treatment of MCF7 cells contained significant overlap with gene signatures derived from breast cancer cell lines that over-expressed EGFR and ERBB2 [24]. This is not surprising as many growth factors activate downstream ERK/MAPK and PI3K/AKT/mTOR signaling but emphasizes that further clinical and laboratory studies will need to be performed to identify if there is a predominant therapeutic target in this subtype of ER+ BC, or if multiple pathways will need to be inhibited to produce clinical benefit in these patients.

The present study also illustrates the ability of generating a biological hypothesis derived from the gene expression profiles of microarray-defined breast cancer molecular subtypes. Heterogeneity of breast cancer clinical behavior and response to tamoxifen has long been apparent to clinicians despite positive expression of the estrogen receptor and its related genes. Studies analyzing ER+ BC prognosis using whole genome microarray data have suggested that ER+ BC is biologically heterogeneous due to the influence of other oncogenic pathways [2-4,6,9]. Thus far however, there has been little research into the pathogenic mechanisms characterizing the individual molecular subtypes apart from the HER2 subtype. Oh and colleagues have proposed abnormal apoptosis and interferon regulation as also contributing to the luminal-B/poor prognostic ER+ BC phenotype [5]. Elucidation of the underlying biological mechanisms is critical for pinpointing an effective therapeutic strategy.

The current data supports the literature on the use of GSEA as a valuable means of generating biological hypotheses utilizing microarray data. A limitation of GSEA is that it relies absolutely on the quality of the gene set that is being analyzed for enrichment and as it uses predefined gene sets, it does not "discover" new information from the magnitude of data available. Other new bioinformatics methods that are being developed will hopefully further our understanding of the underlying oncogenic networks characterizing each of the breast cancer molecular subtypes [25,26].

## Conclusion

In summary, we propose that activation of growth factor signaling contributes to the highly proliferative, relatively tamoxifen-insensitive, luminal-B ER+ BC phenotype and that this signaling is independent of HER2 over-expression. Our study suggests that for this subgroup of ER+ breast cancer patients, targeting this pathway and its upstream mediators could be a useful therapeutic strategy that should be explored in the clinical setting.

## Competing interests

The authors declare that they have no competing interests.

## Authors' contributions

SL and GAM was responsible for the design and execution of the study, collation of study materials, the microarray analysis of study samples, the collection, assembly and verification of the data, data and statistical analysis and interpretation and final manuscript writing; BHK assisted with the microarray analysis of study samples, data analysis and interpretation; FL provided technical support with the microarray analysis; NMC assisted with the western blots; MJP provided the study funding; GAM, TS and CS supervised the study. All authors read and approved the final manuscript.

## Pre-publication history

The pre-publication history for this paper can be accessed here:



## Supplementary Material

Additional file 1 **Growth Factor activation/ERBB2 gene set**. List of Affymetrix probe sets and annotations used for the growth factor/ERBB2 signaling gene set used in the GSEA. Click here for file

Additional file 2 **Table of results generated from Gene Set Enrichment Analysis (GSEA) comparing ER+ BC subtypes**. GSEA comparing low and high-risk ER+ BC subtypes as defined by the GGI. 536 gene sets were assessed for enrichment. Cut-off is taken at a nominal p value < 0.05 and a false discovery rate (FDR) < 0.20. *ES: enrichment score; Norm ES: nomalized enrichment score; p value: nominal p value; FDR: false discovery rate; Color coded pathways: Grey: cell cycle; Pink: metabolism-related; Red: ERBB2/PI3K/AKT-related; Green: apoptosis-related; Blue: DNA damage pathways*. For further information see: Click here for file

Additional file 3 **Ingenuity Pathways Analysis of the growth factor/ERBB2 signaling gene set**. Growth factor/ERBB2 signaling gene set: molecular network proposed by Ingenuity Pathways Analysis (IPA). Top significant canonical pathways is "Neuregulin signaling" (p = 0.005), top related function is "Cancer" (p = 0.00001). Colored nodes represent genes present in the gene set. Click here for file

Additional file 4 **GSEA from MCF-7 cell lines treated with heregulin (HRG)**. Table showing enrichment results for the MCF-7 cell lines treated with heregulin (HRG). Click here for file
