# Supplementary figures and images for: Gene expression profiling identifies activated growth factor signaling in poor prognosis (Luminal-B) estrogen receptor positive breast cancer
Source: BMC Med Genomics. 2009 Jun 24;2:37. doi: 10.1186/1755-8794-2-37 (PMC2706265; doi:10.1186/1755-8794-2-37)

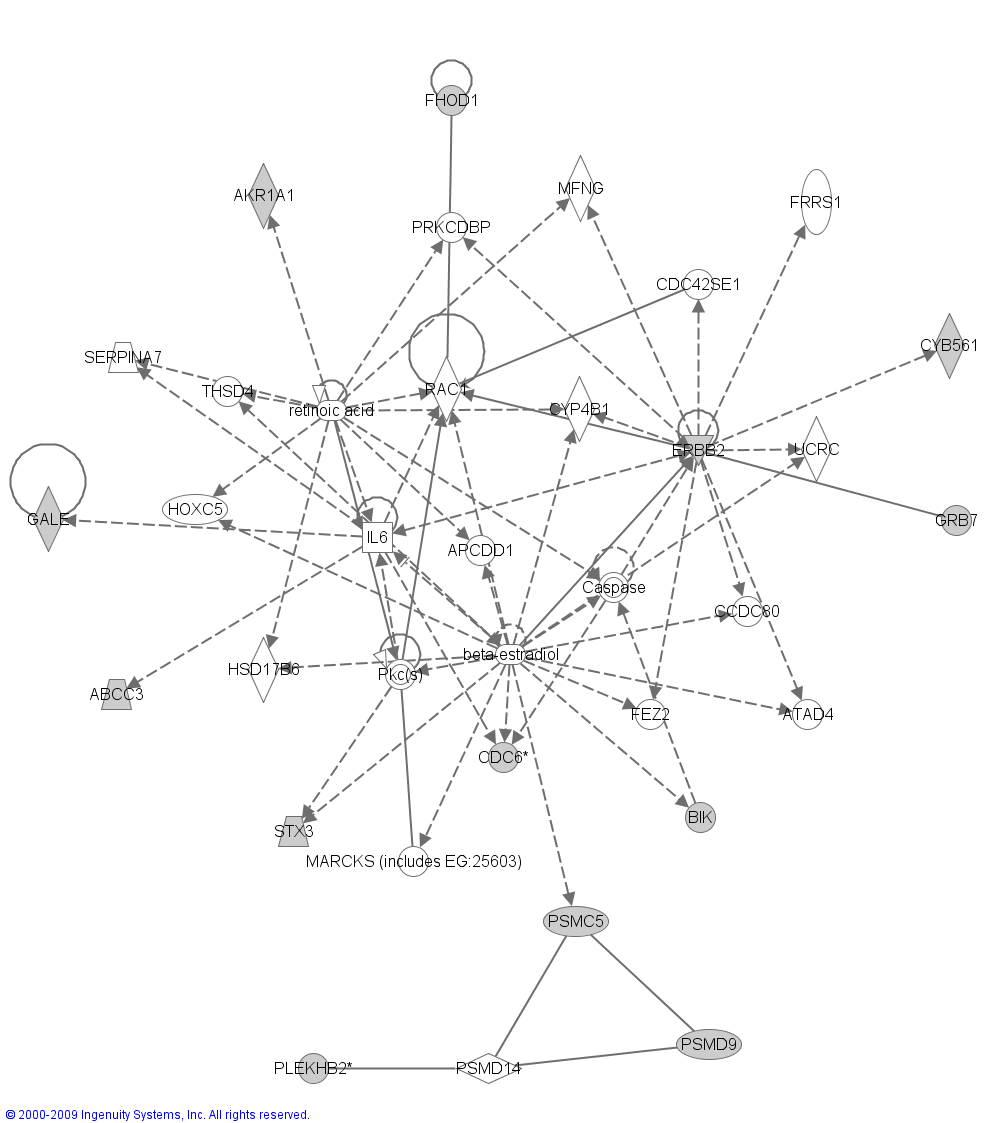

Supplement: Additional file 3 — Ingenuity Pathways Analysis of the growth factor/ERBB2 signaling gene set. Growth factor/ERBB2 signaling gene set: molecular network proposed by Ingenuity Pathways Analysis (IPA). Top significant canonical pathways is "Neuregulin signaling" (p = 0.005), top related function is "Cancer" (p = 0.00001). Colored nodes represent genes present in the gene set. [file 1755-8794-2-37-S3.png]
